# Light-Induced Responses of Slow Oscillatory Neurons of the Rat Olivary Pretectal Nucleus

**DOI:** 10.1371/journal.pone.0033083

**Published:** 2012-03-12

**Authors:** Hanna J. Szkudlarek, Patrycja Orlowska, Marian H. Lewandowski

**Affiliations:** 1 Department of Neurophysiology and Chronobiology, Institute of Zoology, Jagiellonian University, Krakow, Poland; 2 Institute of Physiology I, Westfaelische Wilhelms-University, Muenster, Germany; Virginia Commonwealth University Medical Center, United States of America

## Abstract

**Background:**

The olivary pretectal nucleus (OPN) is a small midbrain structure responsible for pupil constriction in response to eye illumination. Previous electrophysiological studies have shown that OPN neurons code light intensity levels and therefore are called luminance detectors. Recently, we described an additional population of OPN neurons, characterized by a slow rhythmic pattern of action potentials in light-on conditions. Rhythmic patterns generated by these cells last for a period of approximately 2 minutes.

**Methodology:**

To answer whether oscillatory OPN cells are light responsive and whether oscillatory activity depends on retinal afferents, we performed *in vivo* electrophysiology experiments on urethane anaesthetized Wistar rats. Extracellular recordings were combined with changes in light conditions (light-dark-light transitions), brief light stimulations of the contralateral eye (diverse illuminances) or intraocular injections of tetrodotoxin (TTX).

**Conclusions:**

We found that oscillatory neurons were able to fire rhythmically in darkness and were responsive to eye illumination in a manner resembling that of luminance detectors. Their firing rate increased together with the strength of the light stimulation. In addition, during the train of light pulses, we observed two profiles of responses: oscillation-preserving and oscillation-disrupting, which occurred during low- and high-illuminance stimuli presentation respectively. Moreover, we have shown that contralateral retina inactivation eliminated oscillation and significantly reduced the firing rate of oscillatory cells. These results suggest that contralateral retinal innervation is crucial for the generation of an oscillatory pattern in addition to its role in driving responses to visual stimuli.

## Introduction

The pretectal complex is part of the subcortical visual system located in the midbrain. It is divided into five nuclei [1], among which is the olivary pretectal nucleus (OPN). The OPN receives dense retinal innervation [2], [3] and is the first nucleus in the arch of the pupillary light reflex [4]. Neurons within this nucleus can be characterized by low a firing rate in darkness and a tonic ON response following eye illumination. The light-induced response lasts for the duration of the stimulus, and its amplitude is positively correlated with light intensity. This results in a greater activation in the presence of a brighter light [4]–[6].

There is growing evidence that besides its well-established role in pupil constriction [4]–[7], the OPN might also contribute to the regulatory mechanisms of circadian rhythmicity and sleep [8]–[10]. The extensive anatomical connections between the OPN and structures of the circadian clock: suprachiasmatic nucleus - SCN [11], [12] and intergeniculate leaflet - IGL [13]; serve as a base for reciprocal interactions that interfere with circadian behaviors. It was shown that lesions in the pretectal region, or damage to its connections with the IGL, block benzodiazepine-induced phase shift of circadian rhythm [14]. Furthermore, OPN cells are innervated by intrinsically photosensitive retinal ganglion cells (ipRGCs) that govern the entrainment of the circadian clock to external light cues [15]–[18].

Our recent electrophysiological study of OPN cells is particularly interesting in the context of the contribution of the pretectum to the entrainment of circadian rhythms. We characterized a population of OPN neurons that discharge action potentials in a highly rhythmic manner, with an oscillation period of around two minutes [19]. Similar firing patterns have been described for intact networks of the SCN, as well as for the IGL [20], [21]. We have also shown that rhythms in the OPN and ipsilateral IGL are phase synchronized [19]. Although the physiological significance of slow oscillations in circadian system structures has not been determined to date, it is speculated that cells exhibiting such rhythmic firing may underlie the generation of circadian rhythms. This hypothesis is supported by modelling studies assuming that daily rhythm emerge from strong-coupled oscillators [20].

Interestingly, most of the OPN oscillatory cells are located in the shell region of the nucleus [19], which was previously shown to be strongly innervated by ipRGCs [16], [17], [22]. Moreover, cells in the same location show light-induced Fos protein expression which is circadian time dependent and is observed only in response to stimuli presented during the subjective night [8]. Considering these facts, it is interesting to verify whether slow oscillatory OPN cells are light responsive and, if so, whether they constitute a subpopulation of previously characterized luminance detectors or represent a separate group. Our results are based on extracellular recordings from urethane anaesthetized Wistar rats.

## Materials and Methods

### Physiological preparation

Data were obtained from 47 male Wistar rats weighing 270–450 g, bred in our laboratory and kept on a 12∶12 light-dark cycle (lights on at 08:00am). Water and food were available ad libitum. All experiments were carried out in accordance with the European Community Council Directive of 24 November 1986 (86/609/EEC) and in respect of Polish law. The protocol was approved by the Committee on the Ethics of Animal Experiments of the Jagiellonian University (Permit Number: ZI/357/2007). All efforts were made to minimize the number of animals used and to avoid their stress and suffering.

Animals were anaesthetized using urethane (1.5 g/kg dissolved in 2 ml of saline; Sigma) injected intraperitoneally. Both withdrawal and ocular reflexes were checked to ensure stable anaesthesia and additional doses of urethane were given if necessary (10–15% of the initial dose). Body temperature was maintained at 37±0.5°C by using a servo-driven system connected to a heating pad and rectal probe.

Rats were mounted in stereotaxic apparatus (Advanced Stereotaxic Instruments, USA) via ear bars, and the incisor bar was adjusted until the levels of lambda and bregma were equal. Craniotomies were performed either uni- or bilaterally, and dura was removed to enable micropipette penetration. Stereotaxic placement of recording pipettes was conducted for the OPN with bregma as a reference: 4.8 mm posterior, 1.2 mm lateral, and 3.7–4.3 mm ventral to the cortical surface [23].

According to the experimental procedure, one or two borosilicate glass micropipettes (pulled on a horizontal puller; Sutter Instruments, CO P-97, USA) were mounted in a micromanipulator and positioned in the pretectum. The brain surface was flooded with mineral oil. Before electrophysiological recordings, pupils were dilated with a topical application of 1% atropine sulphate solution, and corneas were protected against drying out with mineral oil.

### Recording procedures

Extracellular single cell or multi-unit activities (usually not more than two or three cells) were recorded within the pretectum using fine glass micropipettes filled with 4% Chicago Sky Blue (ChSB; Sigma) in 2 M NaCl (resistance ranging from 5–10 MΩ). Signals were amplified with an Axon Instruments CyberAmp 380 amplifier (Molecular Devices Corporation, USA), recorded on the computer using the Micro mkII interface, and analyzed with Spike2 software (Cambridge Electronic Design Inc., Cambridge, UK). Standardized pulses corresponding to individual action potentials were used for computing on-line frequency time histograms. The data were stored on a computer hard disc for subsequent analysis. An electrical current of −5 µA for 4 minutes was ejected from the recording pipette containing ChSB dye, in order to mark the recording site after completion of the experiment.

**Figure 1:**
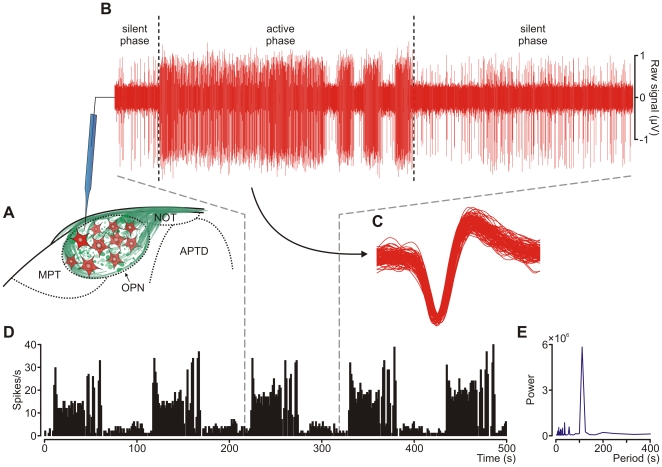
Slow oscillatory activity in the rat OPN. (**A**) Schematic drawing of the pretectal complex depicting neurons (red) located in the OPN and their innervation by axons originating in the retina (green). Dashed curves outline the borders of pretectal nuclei. Some of the OPN neurons (18%) exhibited oscillatory mode of firing (**B**) characterized by two alternating phases of high (active phase) and low (silent phase) activity. Note the characteristic eruptions of the activity prior to the transition to silent phase. (**C**) Waveform sweeps (1.5 ms long) depict one hundred action potentials generated by the oscillatory unit recorded in **B**. (**D**) A firing rate histogram (bin size = 1 second) generated for this unit reflects its rhythmic firing and spectral analysis (**E**) has shown that the dominant periodicity of the rhythm was 110 seconds. APTD, anterior pretectal nucleus, dorsal part; MPT, medial pretectal nucleus; NOT, nucleus of the optic tract; OPN, olivary pretectal nucleus.

All of the experiments were performed on population of OPN cells which generated action potentials in a rhythmic manner, with an oscillation period of approximately two minutes. Firing of these cells was characterized by regular changes from high (active phase) to low (silent phase) activity levels, and a distinct shape could be observed when results were displayed as a frequency time histogram (bin = 1 s, Fig. 1D). Usually, one to six penetrations were required to encounter an oscillatory unit. If the recorded activity did not alter within 3 minutes of the initial penetration, the electrode was advanced until another population of cells was found. Each animal was subjected to only one of the following experiments:

#### Experiment 1: Light-dark-light transitions

Oscillatory activity was recorded in steady-light conditions (baseline activity; 300 lux at the animal eye level) followed by 25 minutes of darkness and a subsequent return to the previous light conditions. The period of oscillation and the mean firing rate for each light condition was calculated and compared with the baseline.

#### Experiment 2: Brief stimulations with light pulses

Prior to electrophysiological recording, the left eyelid of each animal was retracted and white a light-emitting diode (LED) was positioned 3 mm from the left cornea. The LED was used to deliver light flashes. The light spread from the LED covered the entire eye. Flashes were triggered by a Master-8 stimulator (A.M.P.I.; Israel), which was also used to control the duration and intensity of the emitted light. Light intensity was varied by adjusting the LED current, and was calibrated (using averages of 5 measurements) with a digital photometer TES-1336A (TES Electrical Electronic Corp., Taipei, Taiwan). The photometer was positioned 3 mm from the LED, and the following illuminances were used at the cornea): 5, 10, 30, 80, 160, 280, 550 lux. Single-unit recordings were taken from the right OPN under ambient light conditions (100 lux at animal eye level). Immediately after an oscillatory neuron was identified, the light was switched off and the animal was allowed to adapt to darkness for at least 20 minutes. Following the adaptation period, the left eye of the animal was exposed to white light flashes lasting 3 seconds. Light pulses of a given intensity were presented 10 times at 20 second intervals. Neurons were continuously recorded until they were lost or spike discrimination became unreliable. Recordings were rejected if the maximum spike amplitude was less than twice that of the noise range, or if the cell was lost before completion of the tests.

#### Experiment 3: Retina inactivation

Recordings were taken using 300 lux at the animal eye level. After recording a baseline oscillatory activity, the retina in one eye (contra or ipsilateral), was blocked by intravitreal injection of tetrodotoxin (TTX, Sigma; 0.5 M, 10 µl/eye). Following that, TTX was injected into the other eye. TTX applications were performed using a 10-µl Hamilton syringe with a steel needle. The needle was left in place for an additional few minutes before removing it from the eye, in order to ensure proper drug diffusion. A control group was injected with 10 µl of control solution (0.9% NaCl).

### Data analysis

To analyse oscillatory patterns of spike activity we used spectral analysis by applying the Fast Fourier Transform (FFT) algorithm to each consecutive 900 second interval of the rate meter histograms. Both the mean value of the period and the mean firing frequency of the cells were calculated. Changes introduced to these parameters via experimental manipulations (such as turning OFF and turning ON the light, or TTX injections) were analyzed with one-way ANOVA followed by the Tukey post-hoc test. An error probability of *p*<0.05 was considered to be significant.

To determine changes in cell excitation during light flashes, raster plots and peri-stimulus time histograms (PSTH) were calculated. Raster and PSTH displays were 9 seconds long and successive 3 second time periods corresponded to ‘baseline’, ‘stimulation’ and ‘recovery’ states respectively. The amplitude of the response to different light intensities was measured and a mean spike count/100 ms was derived from adequate PSTHs. Statistical comparison (mean ± S.E.M.; repeated measures ANOVA) of the magnitude of cells responses under ‘baseline’, ‘stimulation’ and ‘recovery’ conditions was performed to estimate the strength of activation.

Descriptive statistics are given as mean ± S.E.M. unless stated otherwise. All statistical calculations were performed using Statistica (StatSoft, Inc., USA) and MatLab (MathWorks, Inc., USA).

### Histology

At the end of the experiments the animals were given an overdose of urethane and were perfused transcardially with physiological saline, followed by 4% paraformaldehyde in 0.1 M phosphate buffer (pH = 7.2). The brains were extracted, post-fixed overnight in the same fixative solution and transferred to a 30% sucrose solution in phosphate buffer for cryo-protection. The tissue was frozen and sliced at 50 µm in the coronal plane using a rotary microtome. Sections were mounted on gelatine-coated glass slides. Slices containing ChSB depositions were microscopically inspected to verify recording loci, stained with neutral red, cover-slipped and digitally photographed. The position of the recording pipette tip was plotted onto camera lucida brain drawings with reference to a stereotaxic atlas of the rat brain [23].

## Results

The aim of this study was to provide a comprehensive analysis of light-induced responses of oscillatory neurons and to check the importance of spontaneous retinal activity in the mechanism of the rhythmic firing pattern generation.

**Figure 2:**
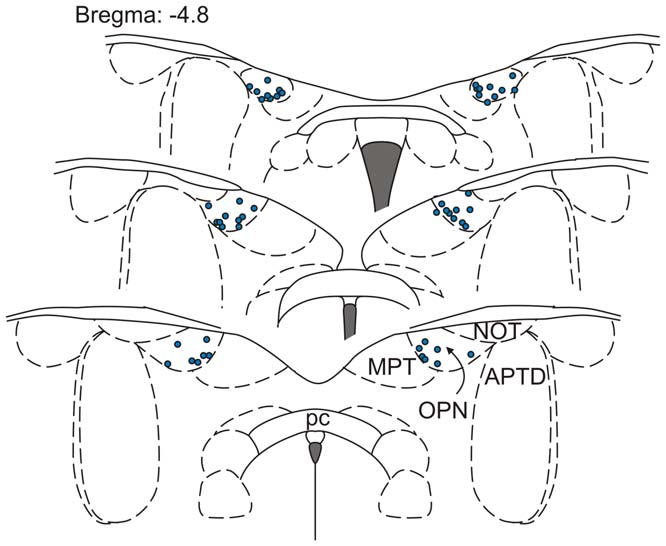
Localizations of recording sites. The schematic position of recording sites is plotted (blue circles) on coronal diagrams from the rat brain atlas [23]. Dashed curves outline the borders of the nuclei. APTD, anterior pretectal nucleus, dorsal part; MPT, medial pretectal nucleus; NOT, nucleus of the optic tract; OPN, olivary pretectal nucleus; pc, posterior commissure.

Extracellular spike activity was measured in a total of 65 recording sites (47 rats, 18 with bilateral recordings). The unifying electrophysiological feature of the recorded cells was their propensity to generate rhythmic firing with a period of approximately two minutes (Fig. 1B and 1D). This was reported in our previous study [19], and is referred to later in the text as slow oscillatory activity (SOA). This rhythmic mode of firing was characterized by two alternating phases: silent and active, with low and high frequency discharges respectively. The transition from the silent to active stage was characterized by a sudden increase of neuronal firing, and the silent phase was preceded by 3 to 4 eruptions of activity intermingled with short lasting inactivations (depicted in Fig. 1A). The oscillatory neurons recorded in this study were histologically verified to be located within the olivary pretectal nucleus (Fig. 2). Based on the results obtained from the 24 animals included in the current study, we estimate that the population of oscillatory cells in the rat constitutes approximately 18% of OPN neurons (cells = 267, oscillatory cells = 47).

### Light-dark-light transitions

#### The effect of ambient light changes on slow oscillatory activity

**Figure 3:**
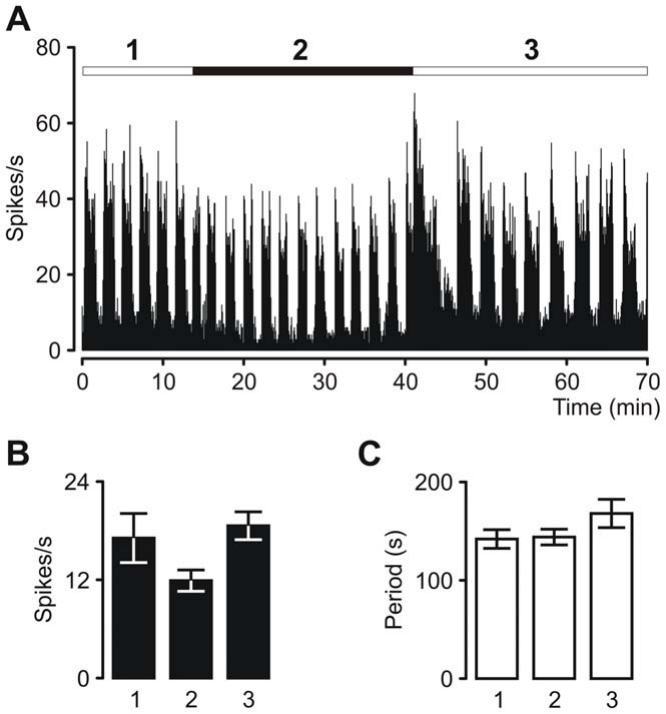
Effects of light-dark-light transitions on oscillatory activity in the OPN neurons. (**A**) Firing rate histogram of a multi-unit recording of OPN oscillatory cells under light-on and light-off conditions. Period of darkness is indicated by black rectangle. Numbers (**1**, **2** and **3**) above the histogram correspond to the baseline light, darkness and recovery of light conditions respectively. (**B**) Average firing rates (per second) and (**C**) mean period of oscillation during different stages. After turning off the light firing rate decreased, however not significantly (n = 14; *p* = 0.27; *F* = 1.33) and the period lengthened after dark-to-light transition (*p* = 0.2; *F* = 1.64). Note, that dark-light transition caused transient disruption of the oscillations.

Based on the fact that rhythmic discharging of IGL cells is light-dependent [21], we checked the influence of light-to-dark and dark-to-light transitions on OPN oscillatory cells in 10 rats (4 out of 10 animals were with bilateral recordings). After recording the baseline SOA the light was turned off (Fig. 3A). Darkness extended over approximately 25 minutes to enable a detailed analysis of dark adaptation on the oscillation period and activity level.

Although dark conditions did not disrupt the oscillatory activity, the average firing rate decreased from 17.1±3.0 Hz to 11.9±2.6 Hz (Fig. 3B). However, this decrease was not statistically significant (*n* = 14; *p* = 0.27; *F* = 1.33). Only in one case did the firing rate decrease almost to zero, but skeleton oscillation could still be observed. The mean period of SOA in darkness was either slightly shorter or did not change at all in comparison to the baseline rhythm. The firing rate at light ON increased to 18.6±3.4 Hz and was comparable to the baseline. A return to the previous lighting conditions resulted in an insignificant prolongation of the rhythm (Fig. 2C) from 142±9.8 s to 167±14.4 s (*p* = 0.20; *F* = 1.64), and the rhythm period did not recover to the baseline. The dark-to-light shift was also accompanied by a transient disruption of oscillation (∼5 minutes) and this stage was characterized by relatively high neuronal firing.

### Brief stimulations with light pulses

#### Evaluation of the illuminance sensitivity of oscillatory OPN cells

Previous electrophysiological reports have shown that OPN neurons code light intensity level [4]–[7]. However, slow oscillatory activities have not been described for these cells. The aim of the conducted experiments was to answer the question whether oscillatory cells have properties similar to luminance detectors in addition to their light responsiveness as shown in *Experiment 1*.

Responses of oscillatory cells to illuminance were investigated by presenting light pulses of different strengths ranging from 5 to 550 lux at the cornea (see Materials and Methods section). Experiments were performed on 18 animals. The statistical analyses are based on 11 single-unit recordings (one cell per animal, Fig. S1). For the remaining 7 experiments, although clear excitatory responses to applied light stimulations and a dependence of the response on stimulus intensity were seen, we were unable to perform accurate spike discrimination. Therefore the amplitude of the responses could not be accurately estimated. This was either due to changes in spike amplitude during the experiment or because cells were lost before the end of the recordings.

**Figure 4:**
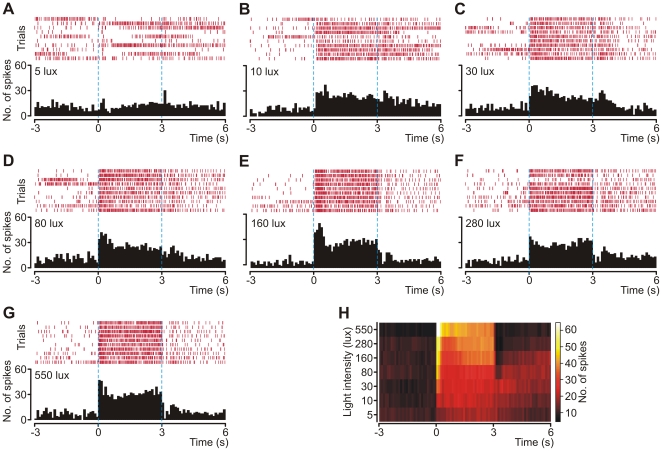
Responses of oscillatory OPN cell to white light pulses of different intensities. Recordings were taken from the right OPN and when oscillatory cell was encountered contralateral eye was stimulated with light pulses of different intensity delivered with white light-emitting diode. Each stimulus lasted for 3 s and given light intensity was presented 10 times at 20 s interval. (**A–G**) Raster displays (top) and peri-stimulus time histograms (bottom) generated for one recorded cell. Each dash in the raster plot corresponds to an action potential and each row to a trial. Trials are time-locked with respect to the onset of stimulus (indicated by time = 0) and stimulus latency is marked with dashed blue line. The activity (summed across all trials of raster display) is shown as frequency histograms, in 100 ms bin width. In the left upper corner of each PSTH light intensity that was used during stimulation is indicated. Note that amplitude of the response increase together with stimulus intensity. (**H**) A composite average PSTH constructed from composite PSTHs obtained for individual light intensity stimulations across all analyzed cells (Fig. S2); the *x*-axis denotes peri-stimulation time, the *y*-axis stimulus strength and neuronal firing (100 ms bin width) is colour-coded (inset on the right side).

**Figure 5:**
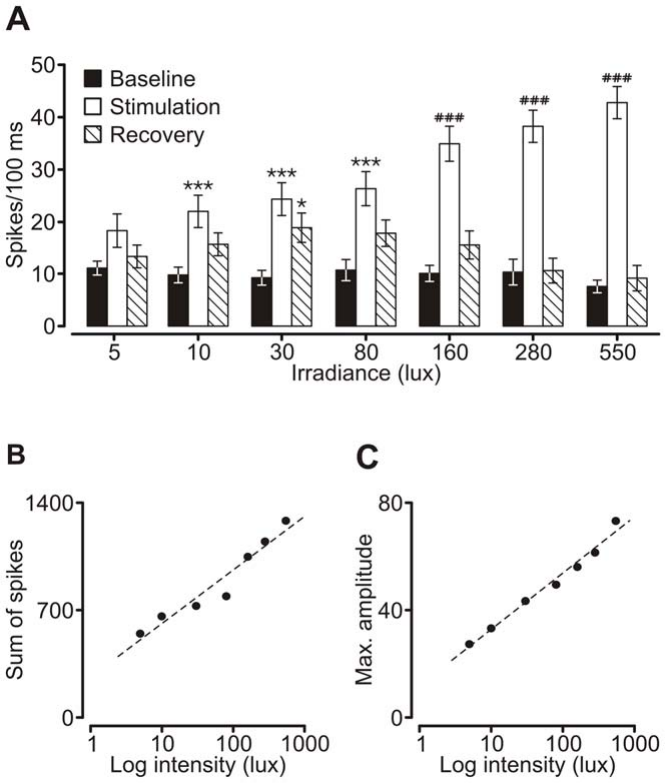
Repeated measures factorial ANOVA analysis of oscillatory neurons responses to light flashes of different intensities. (**A**) Average firing rates during peri-stimulation times of different light intensities (indicated on *x*-axis). Repeated measures factorial ANOVA analysis revealed a highly significant effect of light on neuronal firing (n = 11; *p*<0.00001; *F* = 223.18; Tukey post hoc test; * *p*<0.05, ***p<0.001, ^###^ *p*<0.00005) and strong interaction between the state and light intensities (*p*<0.00001, *F* = 12.38). (**B**) The average sum of spikes generated during the response and (**C**) average maximal amplitude of the transient component of recorded responses to different illumination strength calculated from PSTHs and plotted on semi-logarithmic scale. Note the nearly linear relationship between obtained averages and log of light intensity. Dashed line corresponds to trend-line.

For each analyzed cell, raster plots and corresponding PSTHs (time bin = 100 ms) were computed. A representative example from one of these cells is shown in Fig. 4A–4G. Results demonstrated that contralateral eye illumination evoked an ON response (with the exception of stimulations with 5 lux flashes), characterized by a transient increase at stimulus onset. This was followed by sustained increased activity that lasted for the duration of the stimulus. Some of the neurons (n = 7) displayed transient inhibition lasting approximately 300 ms after cessation of high illuminance stimuli (≥80 lux). An average composite PSTH to demonstrate this results is shown in Fig. 4H, which was computed by averaging the composite histograms obtained for each cell (Fig. S2). The response was found to be stronger when brighter stimuli were used. Additionally, we observed a clear relationship between the maximal amplitude of the transient component of the response (Fig. 5C) and the sum of spikes generated during the response (Fig. 5B) with the logarithmic value of stimulus intensity. Therefore, responses of oscillatory cells were conditioned by light intensity in such a manner that stronger stimuli evoked grater activation of the cells. For the statistical analysis, mean values of ‘baseline’, ‘response’ and ‘recovery’ state derived from PSTHs were subjected to repeated measures factorial ANOVA test followed by Tukey post hoc test. The results showed that responses were significantly different from baseline (*p*<0. 00001, *F* = 223.18; Fig. 5A), and there was a strong interaction between the state (‘baseline’ and ‘response’) and the light intensities used (*p*<0.00001, *F* = 12.38); meaning that one light intensity was activating the cells more than the other.

**Figure 6:**
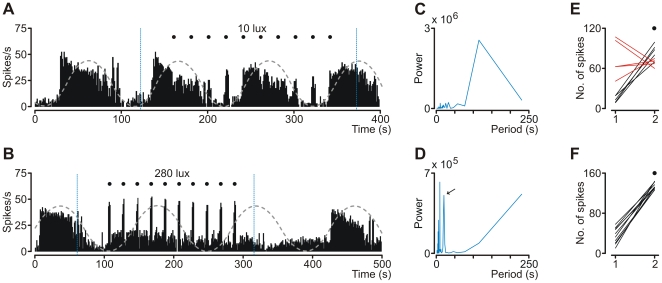
Response profiles of oscillatory cell to a train of light stimuli. (**A**, **B**) Firing rate histograms of oscillatory OPN neuron during train stimulation with 3 second long light flashes (•).Grey dashed sinusoid lines superimposed on histograms reflect baseline oscillation periods. (**A**) The oscillation-preserving response profile was characterized by low responsiveness during active phase of oscillatory cycle and marked augmentation of activity during silent phase of oscillation. Occurrence of this profile was associated with low-intensity light stimulations and does not interrupted slow oscillatory pattern. The oscillation-disrupting response profile (**B**) was characterized by similar responsiveness to each stimulus and was apparent at high-intensity light stimulations. Spectral analysis of the 260 second long segment of the histogram (marked with blue dashed line) using FFT algorithm confirmed oscillation-preserving (**C**) or oscillation-disrupting (**D**) modes of response. Note that the peak location in **C** (115 s) correspond to observed period of oscillation. Plot **D** is lacking the peak at a similar location but instead significant periodicity was detected at 20 second (indicated by an arrow), matching rhythmicity of the stimulation and reflecting responses of the cell. (**E** and **F**) individual light-induced activity changes calculated from respective activity histograms and measured as a number of spikes generated during 3 seconds preceding a stimulus (1) and during a light flash (2). Note that activity changes plotted in **E** are not consistent and when high baseline activity takes place, the response is dampened (red lines).

Our results have also shown a striking peculiarity of the responses to a train of light stimuli of a specific intensity. Two profiles determined by stimuli strength were observed: oscillation-preserving (Fig. 6A) and oscillation-disrupting profile (Fig. 6B). The oscillation-preserving mode was associated with low illuminance stimulations (5, 10 and 30 lux) and was characterized by diminutive, and often inhibitory, responses during the active phase of the SOA and marked enhancement of activity during the silent phase of the SOA (Fig. 6A and 6E). The oscillation-disrupting profile was apparent at light intensities greater than 30 lux. In this mode, each stimulus in the train evoked similar response amplitude (measured as a spike count; shown in Fig. 6B and 6F), and during such train, a slow oscillatory pattern could not be distinguished. Nevertheless, the oscillatory mode of firing was recovered if no subsequent stimuli were applied (Fig. 6B).

### Retina inactivation

#### The effect of retina inactivation with TTX on slow oscillatory activity

After recording the SOA baseline in the OPN, and in order to investigate the relative contribution of retinal afferents to the mechanism responsible for generation of the SOA, we blocked retinal activity using TTX. Experiments were performed on 12 animals (9 out of 12 were with bilateral recordings).

**Figure 7:**
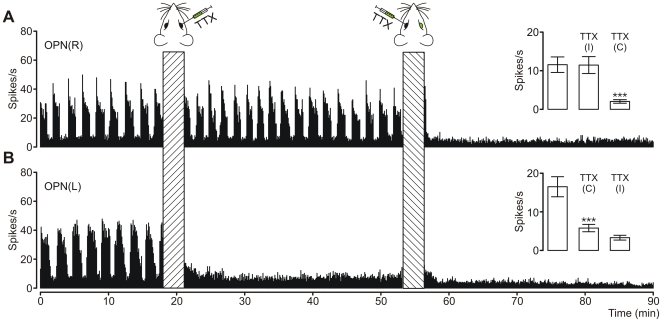
The effect of intraocular injections of TTX on slow oscillatory activities in the OPN. Firing rate histograms of simultaneous recordings of multiunit oscillatory activities in right (OPN(R), **A**) and left (OPN(L), **B**) OPN. After recording baseline activity intravitreal injections (10 µl/eye) of tetrodotoxin were performed. TTX(I) and TTX(C) on the inset graphs denotes injection to ipsilateral and contralateral eye respectively. Note that blockage of contralateral retina (relative to recording site) caused pronounced decrease in firing rate in addition to disappearance of rhythmic spike generation. If contralateral eye was intact, inactivation of ipsilateral retina did not disturb oscillatory pattern. Insets on each plot shows mean ± S.E.M. and results of one-way ANOVA tests (for **A**: n = 12; *p* = 0.0004; *F* = 9.85; for **B**: n = 9; *p* = 0.00001; *F* = 18.19; Tukey post hoc tests; *** *p*<0.001).

Inactivation of the ipsilateral eye (n = 12, Fig. 7A) did not alter the oscillatory firing pattern of OPN cells. However, the subsequent blockage of activity in the contralateral retina completely abolished it (Fig. 7A). Abolition of the cyclic spiking was accompanied by a large decrease in neuronal firing (from 11.5±2.5 Hz to 2.0±0.5 Hz; *p* = 0.0004; *F* = 9.85).

Experiments with the opposite sequence of retinal inactivation (*n* = 9) confirmed the crucial importance of contralateral retinal innervation (Fig. 7B). When the first injection of TTX was applied to the contralateral eye, a 65% decrease in neuronal firing was observed (*p* = 0.00001, *F* = 18.19), alongside a loss of rhythmic spiking. After subsequent inactivation of the ipsilateral retina, a further, insignificant decline in neuronal firing was observed as shown in inset of Fig. 7B.

A control group of 7 animals was injected intravitreally with an equal volume of physiological saline, but no changes in the level of activity were observed (n = 5; *p* = 0.79, *F* = 0.23). However, contralateral saline application was accompanied by an insignificant lengthening of the period from 121±17.4 s to 166±28.1 seconds (*p* = 0.39, *F* = 18.19). This change of the rhythm was not observed in cases of ipsilateral injection.

## Discussion

This study illustrates that ambient lighting conditions are capable of changing major oscillation characteristics of OPN neurons, namely amplitude and period. Oscillatory OPN cells are also able to discriminate between different illuminance levels, revealing similarities with previously described luminance detectors [5], [6]. Moreover, we have shown that rhythmic spike generation by OPN neurons is not an intrinsic property of these cells but a synaptically driven phenomenon.

### Responses to ambient light changes

**Figure 8:**
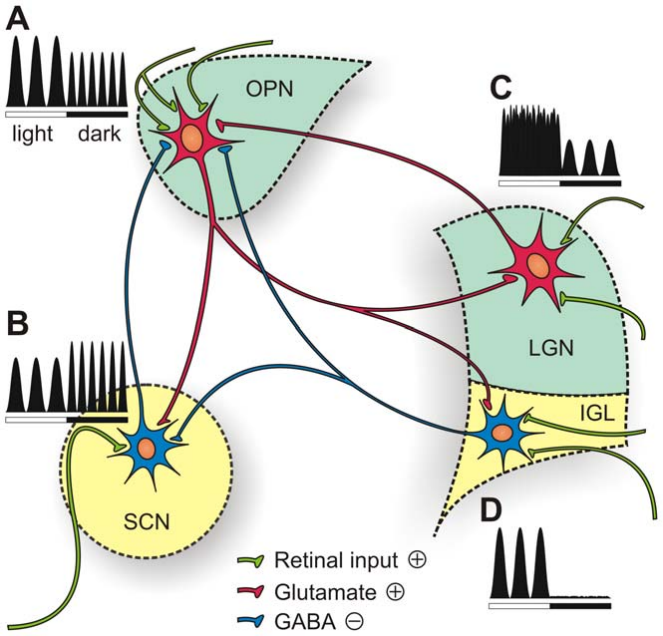
A schematic representation of the modulatory effect of light conditions on oscillatory activities in visual system structures. The coloured drawing illustrates reciprocal connections between the OPN, SCN, LGN and IGL and their innervations originating in the retina. Black and white schemes show slow oscillatory modes of firing and its dependence on light in the OPN (**A**), SCN (**B**), LGN (**C**) and IGL (**D**). Amplitude of the peaks corresponds to activity level and width of the peaks reflects period of oscillations. Light conditions are indicated with rectangles below the schemes: white rectangles correspond to periods of light, black rectangles correspond to darkness. As explained in the text, our assumption is that SOA in the OPN are driven by the retinal excitatory input. Our previous results showed that oscillations in the OPN persist in presence of GABAergic antagonist and therefore, they can not be driven by SCN or IGL neurons, which are GABAergic. On the contrary, the LGN projections are glutamatergic and indirectly convey retinal information to the OPN. Based on the fact that SOA in the LGN occur only in darkness (**B**) it is rather unlikely that LGN drives the rhythm in the OPN because occurrence of the latter one does not depend on the light conditions (**A**).

In our previous study we described a population of OPN neurons characterized by non-stationary activity fluctuating at regular intervals of approximately 140 seconds [19]. Similar slow oscillatory activities were reported for other visual system structures such as the LGN, SCN and IGL. Surprisingly, although very similar in characteristics, slow oscillations in these structures undergo different modulation by ambient light (Fig. 8A–D). In the LGN, cells display oscillation only in darkness, and light causes a switch to tonic firing. As soon as darkness is reintroduced, oscillation recovers [24]. An opposite effect was observed for IGL cells, where oscillations are evident only under light conditions, and darkness almost completely abolishes spiking [21]. In the case of the SCN, oscillations are expressed under both conditions; however, there is a trend of period lengthening when the light is on [20]. Comparing the above data with our results, we noticed that oscillations in the OPN are affected by light changes almost in the same way as slow oscillation in the SCN. First of all, oscillations persist in darkness albeit at a slightly lower amplitude. Secondly, the oscillation period has a tendency to be longer in light conditions. In the case of the OPN, the phenomenon is especially obvious after restoring light conditions, but less evident after the light-to-dark shift (Fig. 3A). If the model of Miller & Fuller [20] is true, it can be assumed that SOAs in the SCN and OPN are responsible for setting the circadian time. This, in turn, influences locomotor activity of the animal. To this respect, a shorter period of SOA in darkness is in accordance with the observation that pulse of darkness presented during the subjective day advances the phase of circadian rhythm [25]–[27].

Perhaps one of the most striking observations in this study was that despite of synchronicity between the OPN and ipsilateral IGL [19], oscillations in the OPN persist after the light is extinguished. We speculate that this divergence between the OPN and IGL are caused by stronger inhibitory inputs affecting oscillatory cells in the IGL. This could be supported by the fact that, in the IGL, firing rate is greatly enhanced after dorsal raphe lesions [28] due to the release from serotonin-mediated inhibition [29]. However, it is not known whether oscillatory activity in the IGL persists in darkness after such lesions.

The unifying feature of slow oscillatory activities in the IGL, SCN and OPN was the transient disruption of the rhythm after transition from dark-to-light. Since neurons constituting the above structures are light responsive [5], [6], [30]–[33], we can assume that the observed transient instability of oscillation reflects period of light adaptation in retinal photoreceptors.

### Irradiance sensitivity

The first described function of the rat OPN was the induction of pupil constriction in response to illumination [4]. Neurons responsible for pupil constriction are described as luminance detectors and possess particular electrophysiological features that allow irradiance coding. That is, low firing rate in darkness and sustained ON response during light stimulation. Illuminance coding is accomplished by a change in response amplitude that gradually increases together with stimulus strength [4]–[6]. Very similar characteristics of the response were obtained for oscillatory cells recorded in the current study (Fig. 5C). Nevertheless, some differences could be observed. Since the firing of neurons recorded in the current study was oscillatory, a low activity level occurred only during the silent phase of oscillation and the recovery time throughout high irradiance train stimulation. However, the most prominent distinction was that responses to low intensity stimulations were locked to the slow phase of oscillation cycles and stimulation during the fast phase sometimes evoked a qualitatively different behavior of the cells (Fig. 6E), namely diminutive inhibition instead of excitation.

Thoss *et al*. [34] have reported that visual sensitivity fluctuates in the same range of frequencies as the oscillatory activity observed in our study. They used subjective threshold light stimuli of 0.1 seconds duration presented at 10 second intervals for sensitivity measures, and observed the responses of pupils to similar flashes but presented in an aperiodic fashion. Moreover, their results of spectral analysis were analogous to ours. Given the fact that an oscillation-preserving response behavior suits sensitivity fluctuations, the conclusion from our experiments is that oscillatory activities may contribute to this phenomenon. In favour of this idea are results showing that the pretectum is crucial for visually-guided behavior as well as visuo-motor reflexes [35]. In addition, OPN neurons project not only to nuclei which mediate pupillary light reflex, but also to the lateral geniculate complex [36], [37], which is decisive for visual perception, or the zona incerta, which is crucial for visual discrimination [38]. Other interpretations are also possible.

Considering the fact that pretectum contributes to REM sleep-triggering in response to the shift from light to darkness [9], [10], it is worthwhile to compare our data with work of Lowenstein *et al*. [39]. This study showed that the pupils of very sleepy people display slow diameter fluctuations that precede sleep initiation. Applying a strong alerting stimulus just before the subject falls asleep restores awaking condition of the subject for some time, and prevents pupil fluctuations. This fact is consistent with our data showing that oscillatory patterns might be interrupted either by transitions from dark-to-light or by train of high irradiance stimuli. In our opinion, oscillatory OPN cells are good candidates for mediating the effects of light on REM sleep.

When attempting to compare our results with previous experiments and trying to reconcile them with pupil diameter adjustments, it is important to bear in mind that elevated neuronal firing in response to eye illumination does not necessarily mean that recorded cells contribute to pupillary light reflex or pupil regulation. It is obvious that recorded oscillatory OPN cells receive excitatory retinal input, but it is not known whether these cells project to the Edinger-Westphal nucleus, which is crucial for launching constriction of the pupil [40]. Luminance-coding properties were also described for the SCN [30], [31] and IGL neurons [32], [33], and up-to-date knowledge shows that these structures do not contribute to pupillary responses.

### Necessity of retinal input

The most important finding of this study was that although oscillations in the OPN were observed under both light and dark conditions, rhythmic spike generation was highly dependent on functional retinal input. This was supported by the fact that after a blockade of sodium conductance in ganglion cells of the contralateral retina, oscillatory activity in the OPN vanishes (Fig. 7). This is also the case in the IGL [41]. Anatomical studies [2], [42] showing that contralateral retinal innervation of the OPN prevails is confirmed by absence of a comparable effect evoked by ipsilateral retina inactivation (Fig. 7A). Although saline injection to the contralateral eye did not abolish oscillations, this procedure resulted in a slight lengthening of the oscillation period. This elongation of the oscillation cycle can be explained by raised eye pressure, which will affects retinal activity [43], [44], and this in turn will result in alterations of activity in all the visual system structures innervated by the retina.

Based on our current results, we suggest that the oscillatory phenomenon in the OPN emerges from excitatory synaptic input from the contralateral retina. This does not exclude the option that the rhythm of the OPN is driven indirectly by other visual system structures such as the IGL, SCN or LGN. However, it is our belief, based on published experimental data, that this is unlikely (Fig. 8). First of all, SOA in the OPN are not blocked by iontophoretic application of GABA antagonist [19], and therefore we can assume that oscillatory activity is driven by an excitatory input. This excludes the hypothesis that SOA are driven by the SCN or IGL, since nearly all of the cells constituting these structures are GABAergic [45], [46]. Another supporting argument against the involvement of the IGL emerges from the fact that slow activity rhythms of IGL cells disappear in darkness [21] while they persist in the OPN (Fig. 3). Since oscillations in the OPN and ipsilateral IGL are phase-synchronized [19], the possibility that the OPN drives oscillation in the IGL might be true. Based on the studies of Albrecht *et al*. [24] it is wrong to assume that glutamatergic input from the LGN is responsible for driving oscillations in the OPN, as SOA in the LGN do not occur under light conditions. Therefore, the most probable excitatory input that impels OPN neurons to fire rhythmically would be the one emerging from retinal ganglion cells, especially as retinal terminals constitute about two thirds of all terminals present in the OPN [47].

Summarizing, our findings fail to support the hypothesis that OPN neurons oscillate independently, suggesting instead that oscillatory phenomena in the OPN arise from complex network interactions. It is possible that the rhythmic discharge of OPN neurons reflects oscillation in the afferent retinal input. This hypothesis might be supported by the fact that the oscillatory behaviour of retinal ganglion cells has been described in developing ferret retina [48] and in the retina of an adult cat [49].

### General conclusions

Slow oscillatory neuronal activities have attracted growing interest due to their possible involvement in the processes of attention [50], stress responses [51], regulation of arousal [52] or circadian rhythms [20], [21]. The presence of slow rhythms suggests their physiological role, though their exact function is not clearly delineated and can be only speculated.

From our current experiments it is evident that regular fluctuations in the firing rate of OPN neurons are involved in the processing of acute and ambient visual information. Responses to specific acute stimuli are phase-locked and, therefore, they might have an unambiguous role in sensory integration while visual information about ambient light level is necessary for driving the rhythm and adjusting its amplitude and period. Due to the similarities observed between oscillatory phenomena in the OPN, IGL or SCN, anatomical connections between these structures [11]–[13], [36], [53] and the dependence of slow rhythm expression on retinal input (at least in case of OPN and IGL), we speculate that oscillations in above mentioned structures are driven by melanopsin retinal ganglion cells. This is supported by the fact that these nuclei are specifically innervated by melanopsin cells [15]–[18], and that internal calcium fluctuations in some melanopsin cells, which probably reflect neuronal activity, persist in darkness [54]. Nevertheless, the above hypothesis requires further investigation.

## Supporting Information

Figure S1 **Slow oscillatory activity of OPN neurons.** Firing rate histograms (bin size = 1 second) of the 11 OPN neurons that were subjected to *Experiment 2*. Their respective responses to light stimulations are depicted in Fig. S2. The *x*-axis denotes time (s) and the *y*-axis denotes firing rate (Hz).
(TIF)

Figure S2 **Responses of oscillatory OPN cells to white light pulses of different intensities.** Composite PSTHs were computed for every cell tested (n = 11) derived from PSTHs obtained during stimulations at individual light intensity. The *x*-axis denotes peri-stimulation time, and the *y*-axis indicates stimulus strength. The neuronal firing (100 ms bin width) is colour-coded (inset on the right side). A composite PSTH corresponding to the cell shown in Fig. 4, is displayed in the first row of the third column.
(TIF)
