# Supplementary figures and images for: Light-Induced Responses of Slow Oscillatory Neurons of the Rat Olivary Pretectal Nucleus
Source: PLoS One. 2012 Mar 12;7(3):e33083. doi: 10.1371/journal.pone.0033083 (PMC3299748; doi:10.1371/journal.pone.0033083)

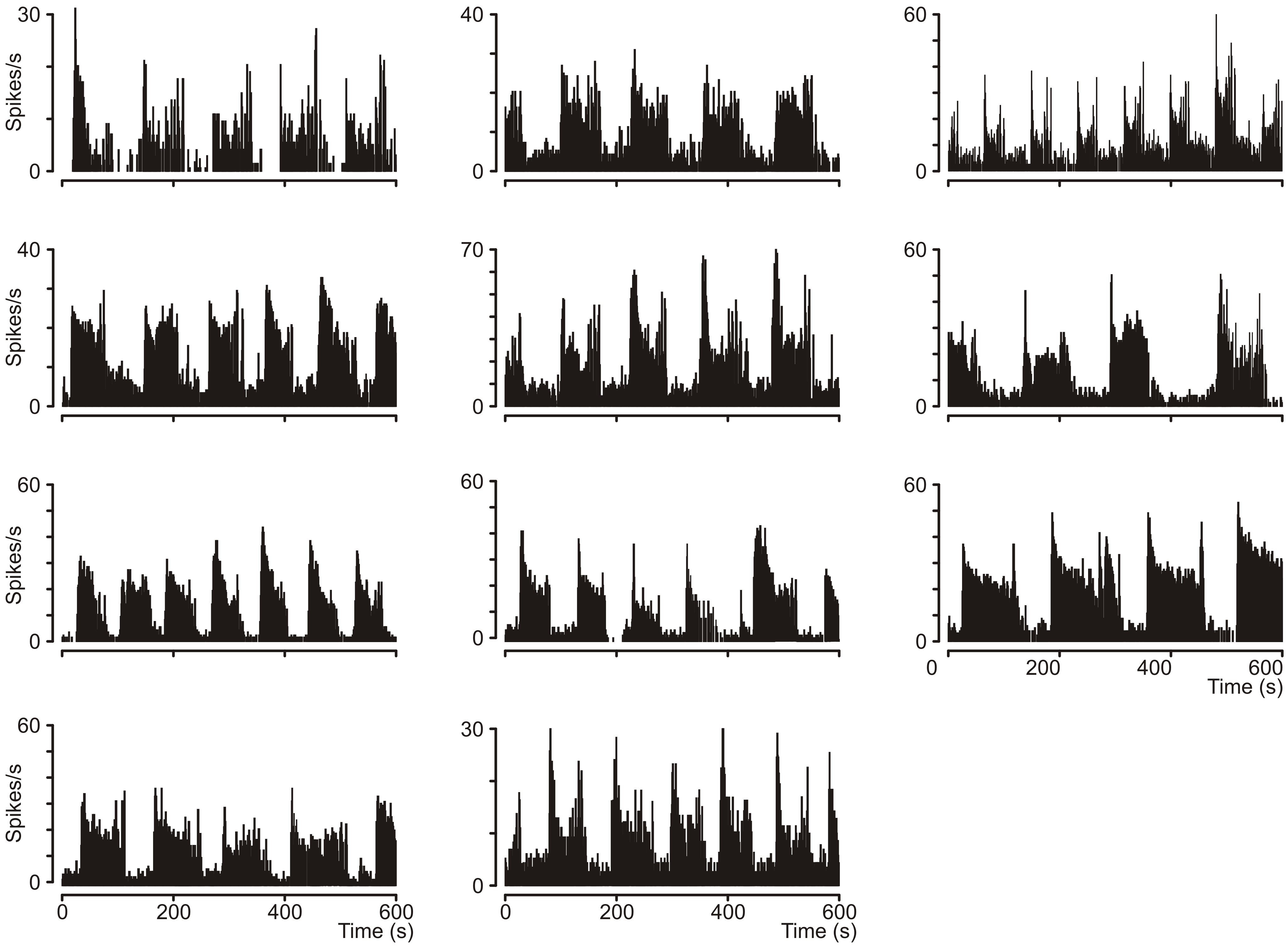

Supplement: Figure S1 — Slow oscillatory activity of OPN neurons. Firing rate histograms (bin size = 1 second) of the 11 OPN neurons that were subjected to Experiment 2. Their respective responses to light stimulations are depicted in Fig. S2. The x-axis denotes time (s) and the y-axis denotes firing rate (Hz). (TIF) [file pone.0033083.s001.tif]

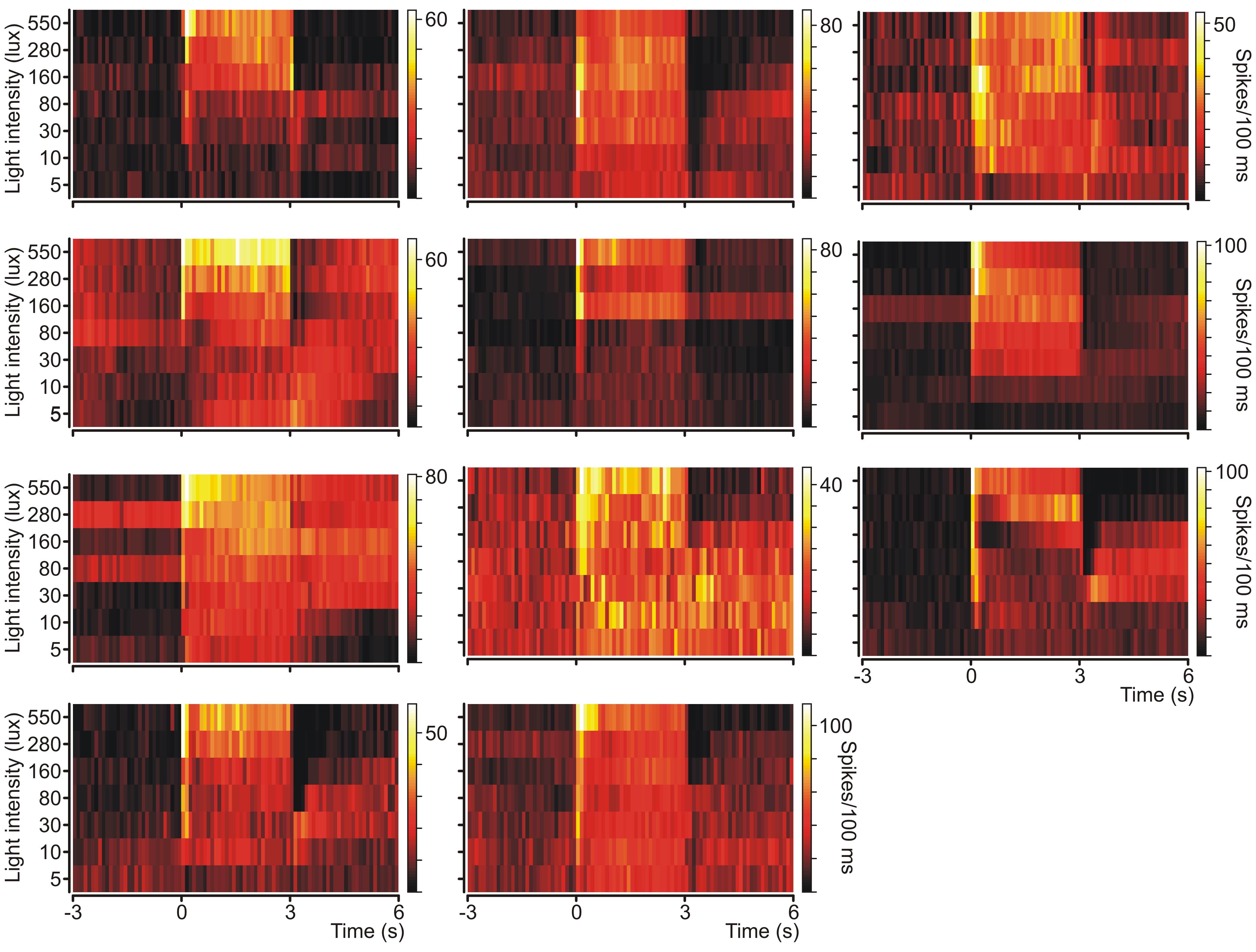

Supplement: Figure S2 — Responses of oscillatory OPN cells to white light pulses of different intensities. Composite PSTHs were computed for every cell tested (n = 11) derived from PSTHs obtained during stimulations at individual light intensity. The x-axis denotes peri-stimulation time, and the y-axis indicates stimulus strength. The neuronal firing (100 ms bin width) is colour-coded (inset on the right side). A composite PSTH corresponding to the cell shown in Fig. 4, is displayed in the first row of the third column. (TIF) [file pone.0033083.s002.tif]
